# Identification of Deregulated Pathways, Key Regulators, and Novel miRNA-mRNA Interactions in HPV-Mediated Transformation

**DOI:** 10.3390/cancers12030700

**Published:** 2020-03-16

**Authors:** Iris Babion, Viktorian Miok, Annelieke Jaspers, Angelina Huseinovic, Renske D. M. Steenbergen, Wessel N. van Wieringen, Saskia M. Wilting

**Affiliations:** 1 Pathology, Cancer Center Amsterdam, Amsterdam UMC, Vrije Universiteit Amsterdam, 1081 HV Amsterdam, The Netherlands; i.babion@amsterdamumc.nl (I.B.); viktormiok@gmail.com (V.M.); a.jaspers@amsterdamumc.nl (A.J.); a.huseinovic@amsterdamumc.nl (A.H.); 2 Epidemiology & Biostatistics, Amsterdam Public Health, Amsterdam UMC, Vrije Universiteit Amsterdam, 1081 HV Amsterdam, The Netherlands; w.vanwieringen@amsterdamumc.nl; 3 Department of Functional Sciences, Faculty of Medicine, Victor Babeş University of Medicine and Pharmacy of Timişoara, 300041 Timişoara, Romania; 4 Department of Mathematics, VU University Amsterdam, 1081 HV Amsterdam, The Netherlands; 5 Department of Medical Oncology, Erasmus MC Cancer Institute, Erasmus University Medical Center, 3015 GD Rotterdam, The Netherlands; s.wilting@erasmusmc.nl

**Keywords:** microRNA, mRNA, CGH, cervical cancer, HPV, microarray, TGF-beta, PITX2

## Abstract

Next to a persistent infection with high-risk human papillomavirus (HPV), molecular changes are required for the development of cervical cancer. To identify which molecular alterations drive carcinogenesis, we performed a comprehensive and longitudinal molecular characterization of HPV-transformed keratinocyte cell lines. Comparative genomic hybridization, mRNA, and miRNA expression analysis of four HPV-containing keratinocyte cell lines at eight different time points was performed. Data was analyzed using unsupervised hierarchical clustering, integrated longitudinal expression analysis, and pathway enrichment analysis. Biological relevance of identified key regulatory genes was evaluated in vitro and dual-luciferase assays were used to confirm predicted miRNA-mRNA interactions. We show that the acquisition of anchorage independence of HPV-containing keratinocyte cell lines is particularly associated with copy number alterations. Approximately one third of differentially expressed mRNAs and miRNAs was directly attributable to copy number alterations. Focal adhesion, TGF-beta signaling, and mTOR signaling pathways were enriched among these genes. PITX2 was identified as key regulator of TGF-beta signaling and inhibited cell growth in vitro, most likely by inducing cell cycle arrest and apoptosis. Predicted miRNA-mRNA interactions miR-221-3p_BRWD3, miR-221-3p_FOS, and miR-138-5p_PLXNB2 were confirmed in vitro. Integrated longitudinal analysis of our HPV-induced carcinogenesis model pinpointed relevant interconnected molecular changes and crucial signaling pathways in HPV-mediated transformation.

## 1. Introduction

Development of cervical cancer is a multi-step process initiated by a persistent infection with a high-risk type of the human papillomavirus (HPV) [1]. Following infection of the basal epithelial cells by HPV, a productive infection is established, which is characterized by the production of new viral particles [2,3]. The expression of viral proteins is tightly linked to host cell differentiation and is necessary for viral genome replication in differentiated cells. Aberrant expression of viral oncogenes E6 and E7 in proliferating cells, however, leads to the abortion of the viral life cycle and triggers malignant transformation. While E6 and E7 initiate and maintain transforming infections, additional molecular changes in the host cell genome are required for the development of invasive cancer.

Molecular alterations associated with cervical carcinogenesis are of both genetic and epigenetic nature and ultimately lead to aberrant expression of oncoproteins and tumor suppressors. Genome-wide analyzes of cervical tissue specimens have led to the identification of numerous chromosomal aberrations and differentially expressed coding and non-coding genes in cervical (pre)cancer [4,5,6]. As the sequential order of molecular changes as well as their causative relevance for cancer development cannot be extrapolated from cross-sectional data, it has proven problematic to distil promising disease markers and potential therapeutic targets from these observations.

In vitro cell line models of HPV-mediated transformation, in which primary keratinocytes are transfected with HPV16 or HPV18, have been shown to faithfully mimic cervical cancer development [7,8,9]. This offers the unique opportunity to study (epi)genetic alterations during carcinogenesis. Integrative analysis of longitudinal data obtained from multiple molecular levels allows for reconstruction of the sequential order in which molecular changes occur and is likely to result in the identification of crucial molecular alterations that drive the carcinogenic process. Importantly, HPV-transformed keratinocytes have previously been shown to provide useful model systems to study chromosomal instability in cancer in general [10], indicating that insights in HPV-induced transformation might not only provide a better understanding of cervical cancer development, but could be applicable to other cancers, too.

**Figure 1:**
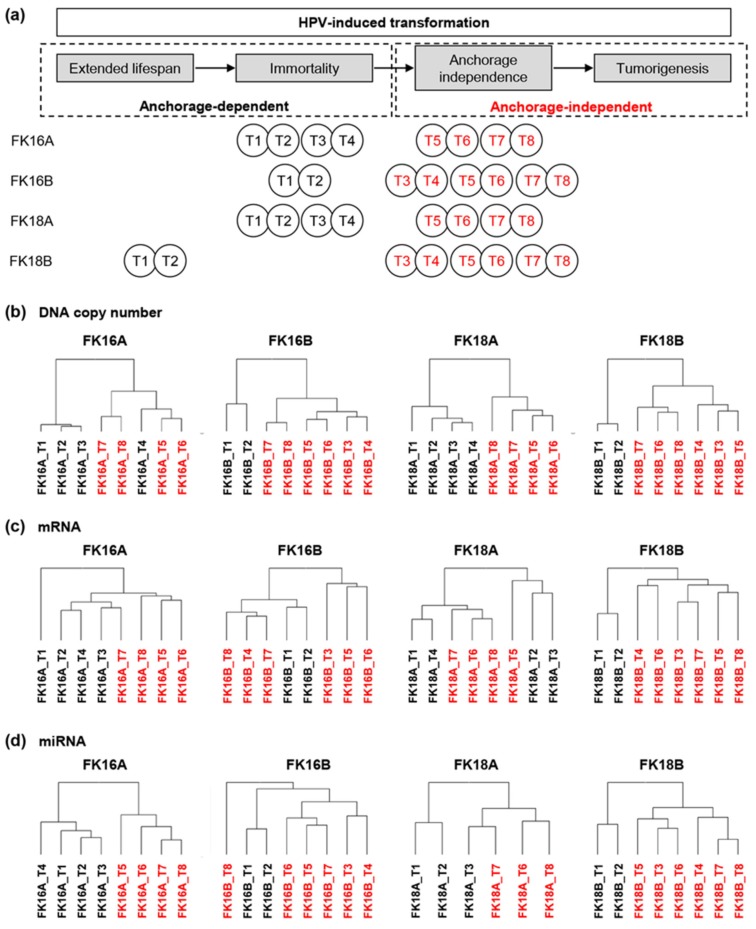
Characterization of our longitudinal in vitro model system of human papillomavirus (HPV)-induced transformation. (**a**) Anchorage-dependent (black) and -independent (red) time points (T) of all four cell lines are shown in relation to the transformation process [16]. MiRNA microarrays of cell line FK18A at T4 and T5 did not pass quality control and were therefore excluded from miRNA analysis. Unsupervised hierarchical cluster results based on (**b**) DNA copy number, (**c**) overall mRNA expression, and (**d**) overall miRNA [16] expression are shown for FK16A, FK16B, FK18A, and FK18B.

Previous studies have demonstrated that HPV-induced transformation can be divided into four stages (Figure 1a) [11,12]: an extended lifespan (1) is acquired as a result of E6 and E7 mediated inhibition of tumor suppressor genes TP53 and RB1 [13]. Genetic instability induced by E6 and E7 subsequently generates an immortal phenotype (2) usually associated with the activation of the telomerase enzyme resulting from upregulation of TERT. During prolonged culturing of HPV-immortalized keratinocytes additional (epi)genetic alterations accumulate that can eventually lead to anchorage-independent growth (3), considered as proof for complete transformation in vitro [14,15], and tumorigenicity (4).

Here, we present the first comprehensive molecular profiling of HPV-induced carcinogenesis over time. DNA copy number changes, mRNA, and miRNA expression were determined in four individual HPV-transformed keratinocyte cell lines at eight different passages representing different stages of HPV-induced transformation (hereafter referred to as time points). Integrative temporal analysis of this unique longitudinal multi-level dataset allowed for the identification of relevant pathways and associated key regulators as well as the prediction of miRNA-mRNA interactions in HPV-induced transformation. The obtained results will greatly accelerate the identification of those molecular alterations that are crucial to HPV-induced carcinogenesis and as such will likely provide highly specific and sensitive disease markers. These are urgently needed to improve current cervical cancer screening programs for the coming era, especially with the implementation of HPV-based screening and HPV vaccination in more and more countries.

## 2. Results

### 2.1. Anchorage Independence Coincides with Marked Molecular Changes

To study the molecular events driving HPV-induced transformation, we investigated four independent HPV-transformed keratinocyte cell lines at different stages during transformation. Copy number changes, mRNA, and miRNA expression were determined in all four cell lines at six to eight different passages (time points) (Table 1). As described previously, the selected time points represent distinct stages in HPV-induced transformation (Figure 1a) [16].

To obtain an unbiased overview of the observed molecular changes over time in the four cell lines, we performed unsupervised clustering analysis of genomic as well as mRNA and miRNA expression profiles. Chromosomal profiles of all four cell lines were separated almost perfectly based on their ability to grow anchorage-independently (Figure 1b), which supports our previous observation based on miRNA expression profiles (Figure 1d) [16]. Revealing a slightly less clear-cut separation, a sub-cluster enrichment for either anchorage-dependent or anchorage-independent time points was still noticeable for mRNA profiles (Figure 1c).

### 2.2. Approximately One Third of Differentially Expressed Genes is Associated with Copy Number Changes

To identify genes relevant to HPV-induced carcinogenesis, we performed integrative longitudinal differential gene expression analysis, which also takes the genomic copy number into account [17]. In total, expression of 3642 mRNA genes and 106 mature miRNAs (corresponding to 118 miRNA genes) was found to either increase or decrease consistently over time in at least three out of four cell lines analyzed (Table S1 and S2). An association with copy number was observed in 33.9% of significant mRNAs (1233 genes, Table S1) and 32.2% of miRNA genes (38 genes, Table S2).

A technical validation of our microarray results was performed by qRT-PCR for three mRNAs (DEK, DKK3, SLC25A36, Figure S1a) and five miRNAs (miR-100-5p, miR-103a-3p, miR-125b-5p, miR-15b-5p, miR-221-5p, Figure S1b). Moreover, in vivo relevance of our findings was investigated for those differentially expressed mRNAs and miRNAs for which in-house microarray data of cervical tissue samples was available (mRNAs: 2661 out of 3642, miRNA: 39 out of 106). A concordant significant pattern of up- or downregulation was observed from normal to CIN to SCC by Spearman correlation for 24% of mRNAs and 49% of miRNAs, whereas 15% of mRNAs and 18% of miRNAs exhibited an opposite expression pattern in tissues and the cell line model (Tables S1 and S2).

### 2.3. Pinpointing Key Regulators in Enriched Pathways

**Figure 2:**
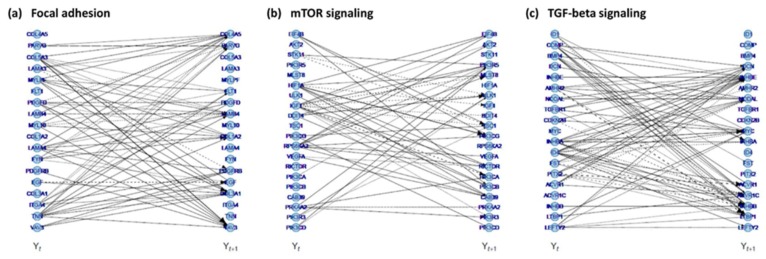
Pathway reconstruction. Inferred dynamic networks of the (**a**) focal adhesion, (**b**) mTOR signaling, and (**c**) TGF-beta signaling pathways. Blue circles are the nodes of the network and represent genes. The lines depict which genes’ mRNA expression level at the current time point, denoted by Y_t_, affect the genes’ mRNA expression level at a future time point, denoted Y_t + 1_. Solid and dashed lines indicate whether this relation is of a stimulating (positive) or suppressing (negative) nature, respectively. For plotting purposes, genes with a total edge strength (sum of all absolute in and out connections of the respective gene) <0.25 (or <1 for TGF-beta signaling) were excluded.

Based on the unsupervised hierarchical cluster results we hypothesized that a substantial proportion of the above described DNA copy number induced gene expression changes are involved in the acquisition of anchorage independence. Indeed, three of the most significantly overrepresented pathways among all DNA copy number associated genes (Table S3), namely focal adhesion (KEGG hsa04510), TGF-beta (KEGG hsa04350), and mTOR signaling (KEGG hsa04150), are implicated in processes underlying anchorage independence (i.e., anoikis resistance and induction of EMT) [18]. To further investigate this notion, we translated our longitudinal data into pathway-based networks for focal adhesion, TGF-beta signaling, and mTOR signaling (Table S3 and Materials and Methods section). Data-driven longitudinal networks were built for all genes in the respective pathways taking copy number into account and key regulators were identified (Table 2, Figure 2, Table S4).

### 2.4. TGF-Beta Pathway

**Figure 3:**
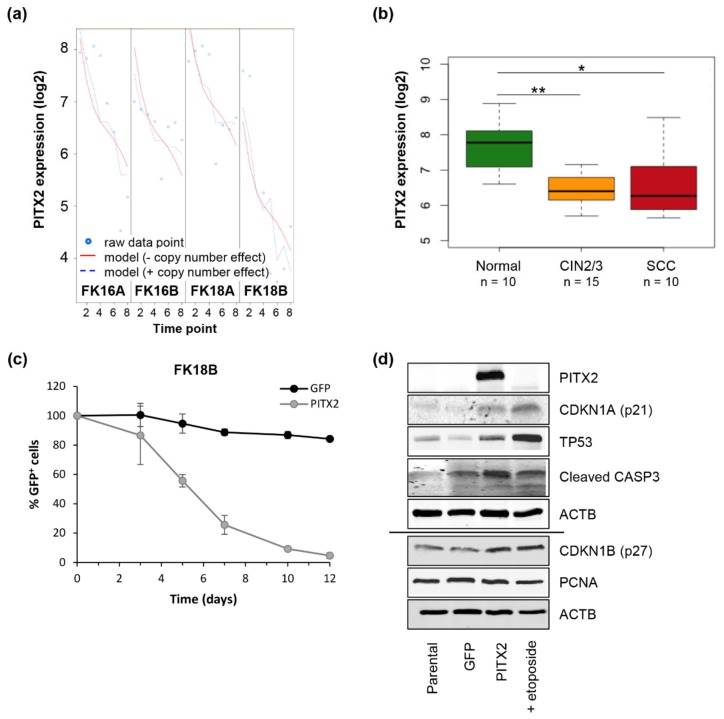
PITX2 acts as tumor suppressor in HPV-induced transformation. (**a**) PITX2 expression in HPV-transformed keratinocyte cell lines. Using our tigaR method, a model without (red continuous line) and with copy number effect (blue discontinuous line) was fitted [17]. Similarity between the fitted models indicates that differential PITX2 expression is not copy number-driven. (**b**) PITX2 expression in mRNA microarray data obtained from normal HPV-positive cervical epithelium (Normal), high-grade precancerous lesions (CIN2/3), and squamous cell carcinomas (SCC). Boxplots show medians with lower and upper quartiles, and range whiskers. * *p* < 0.05, ** *p* < 0.005, according to the Wilcoxon rank-sum test. (**c**,**d**) Late FK18B cells (ca. passage 190) were transduced with an empty LeGO-iG2 vector or a LeGO-iG2-PITX2 construct. (**c**) FACS analysis: Mean and standard deviation of two independent experiments are shown. (**d**) Western blot analysis. Cells were harvested 72 h after transduction. Cells treated with etoposide were included as positive control for TP53/CDKN1A signaling.

TGFB1 has previously been shown to act as tumor suppressor during early stages of cervical carcinogenesis, although it takes on a tumor promoting function at a later stage [19]. Here, we identified PITX2 as key regulator of TGF-beta signaling (Table 2c). PITX2 was downregulated in all four keratinocyte cell lines (Figure 3a) and this was concordant with our in-house tissue data (normal to CIN2/3 to SCC, Figure 3b).

To investigate the functional relevance of PITX2, a PITX2 expression vector was transduced in late FK18B cells. Cell growth analysis demonstrated a decrease in cell viability after three days of PITX2 overexpression compared to empty vector (GFP only) transduced FK18B cells (Figure 3c,d). PITX2 has previously been shown to cause G0/G1 arrest in HPV18-positive HeLa cells through accumulation of TP53 and CDKN1A (p21) [20,21]. In line with this, we found elevated TP53 and CDKN1A protein levels in PITX2 overexpressing FK18B cells (Figure 3d). To elucidate whether the observed growth reduction in PITX2 overexpressing cells is caused by TP53/CDKN1A-mediated cell cycle arrest or apoptosis, we further analyzed known marker proteins CDKN1B (p27), PCNA, and cleaved CASP3. Even though there was no change in PCNA levels upon PITX2 overexpression, increased levels of CDKN1B and cleaved CASP3 suggested that reduced cell viability in PITX2 overexpressing cells is associated to both cell cycle arrest and apoptosis (Figure 3d).

### 2.5. Identification of Potential miRNA-mRNA Target Interactions

Using our developed tigaR framework we next investigated the relation between miRNA and mRNA expression in a longitudinal fashion. For this analysis, we only included miRNAs for which altered expression was not contradictory to the pattern observed in the tissue specimens (n = 86). When restricting the analysis to miRNA-mRNA pairs showing significant expression changes over time in the opposite direction, 634 interactions between 27 miRNAs and 372 mRNAs were identified (Table S5). For the verification of the 634 potential interactions, we used three up-to-date publicly available target prediction databases: RNA22 v2.0, miRDB v5, and TargetScan v7 [22,23,24]. Four interactions were predicted by all three databases (Table S5).

**Figure 4:**
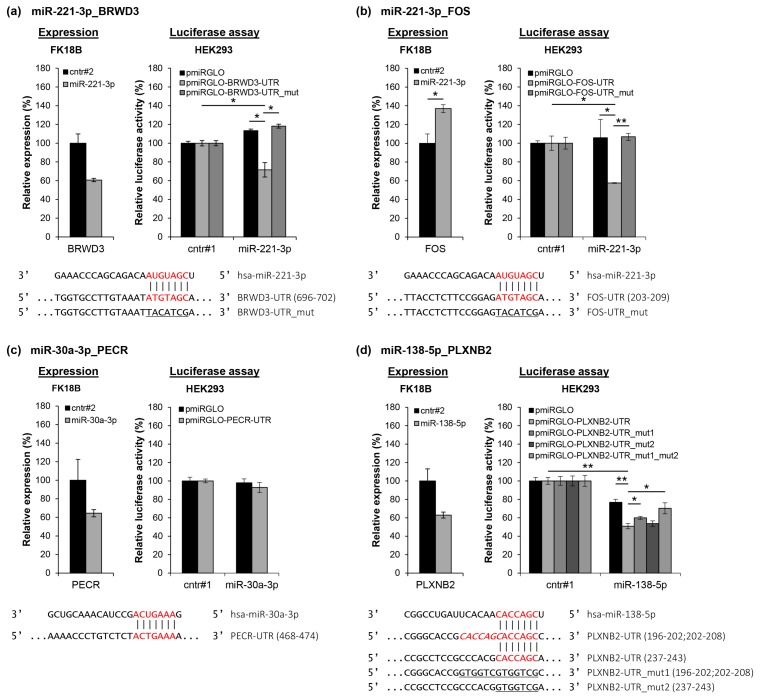
Confirmation of miRNA-mRNA interactions. Predicted miRNA-mRNA interactions (**a**) miR-221-3p_BRWD3, (**b**) miR-221-3p_FOS, (**c**) miR-30a-3p_PECR, and (**d**) miR-138-5p_PLXNB2 were investigated using mRNA expression analysis and dual-luciferase reporter assays. Left panel: Effects of ectopic expression of the miRNA on the expression level of its predicted mRNA target. Expression levels were determined by qRT-PCR in late passage FK18B cells (ca. passage 190) transfected either with a negative control (cntr#2) or with the respective miRNA mimic. Data was normalized to SNRPA. Mean and standard deviation of two technical replicates are shown. Right panel: Dual-luciferase reporter assay to confirm direct interaction between the miRNA and its predicted target mRNA. HEK293 cells were transiently transfected with a negative control (cntr#1) or the respective miRNA mimic in combination with either an empty pmiRGLO vector, a pmiRGLO construct containing the predicted binding site (pmiRGLO-predicted target-UTR), or a pmiRGLO construct containing a mutated binding site (pmiRGLO-predicted target-UTR_mut). MiRNA, UTR, and binding sites are indicated in the lower panel. Mean and standard deviation of three technical replicates are shown. * *p* < 0.05, ** *p* < 0.005., according to the two-sided Student’s *t*-test.

To validate our miRNA target prediction, the four miRNA-mRNA interactions that were predicted by all three databases, i.e., miR-138-5p_PLXNB2, miR-221-3p_BRWD3, miR-221-3p_FOS, and miR-30a-3p_PECR, were selected. For all four miRNA-mRNA pairs, mRNA expression increased and miRNA expression decreased with increasing passage number in at least three out of four cell lines. Ectopic overexpression of the respective miRNA in late passage FK18B cells resulted in reduced expression levels of the predicted mRNA targets BRWD3, PECR, and PLXNB2 (Figure 4a–d). Ectopic overexpression of miR-221-3p surprisingly led to 40% increase of FOS expression in FK18B.

Next, dual-luciferase assays were performed to investigate whether the effects of miRNAs on their target mRNAs are the result of direct interactions. As shown in Figure 4a, co-transfection of pmiRGLO-BRWD3-UTR and ectopic miR-221-3p in HEK293 cells decreased luciferase activity compared to either co-transfection of pmiRGLO-BRWD3-UTR with a non-targeting control sequence or the empty pmiRGLO vector with ectopic miR-221-3p. Directed mutagenesis of the predicted miR-221-3p seed sequence (pmiRGLO-BRWD3-UTR_mut) abolished the reduction in luciferase activity observed with the wild type vector (Figure 4a). Likewise, a direct interaction between miR-221-3p and the cFOS 3’UTR could be demonstrated (Figure 4b). Luciferase activity of a pmiRGLO-PECR-UTR construct was comparable to that of the empty pmiRGLO vector in the presence of ectopic miR-30a-3p or a non-targeting control sequence, suggesting that the observed reduction in PECR mRNA levels upon ectopic overexpression of miR-30a-3p is an indirect effect (Figure 4c). Interestingly, PLXNB2 has two predicted miR-138-5p binding sites in the 3’UTR. Luciferase activity of the pmiRGLO-PLXNB2-UTR vector was reduced in the presence of miR-138-5p compared to the empty vector (Figure 4d). Mutation of the first binding site significantly restored luciferase activity, while mutation of the second binding site alone had almost no effect. The largest and most significant restoration of luciferase activity was obtained when both binding sites were mutated.

**Figure 5:**
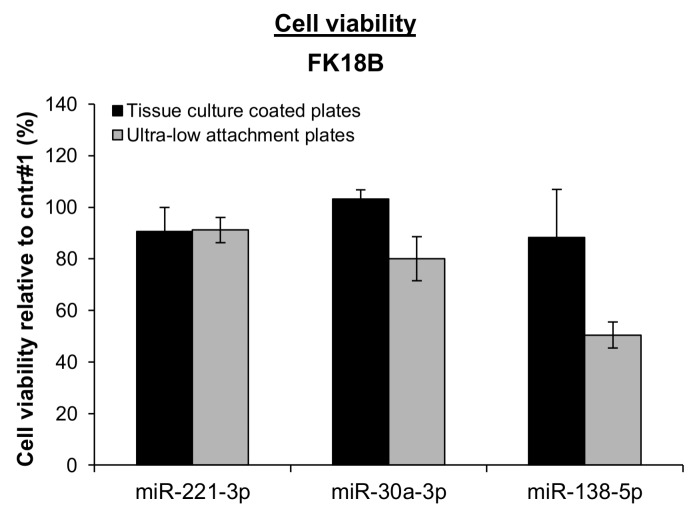
Effect of miRNAs on adherent and anchorage-independent growth of late passage FK18B cells. Cell viability of late passage FK18B cells upon ectopic expression of miR-221-3p, miR-30a-3p, and miR-138-5p was measured in adherent tissue culture coated plates and ultra-low attachment plates and standardized against miRNA mimic negative control #1. Mean and standard errors of three independent experiments are shown.

Interestingly, ectopic expression of miR-30a-3p and miR-138-5p decreased anchorage-independent growth of late passage FK18B cells as measured on ultra-low attachment plates (Figure 5). No reduction in cell viability was observed on normal, adherent plates, suggesting involvement of these miRNAs specifically in anchorage independence.

## 3. Discussion

We here present one of the most extensive studies characterizing HPV-induced transformation in a longitudinal fashion. Unsupervised hierarchical clustering of our copy number data separated nearly perfectly between anchorage-dependent and -independent passages of HPV-transformed cells, demonstrating the importance of chromosomal alterations in the acquisition of anchorage independence. Moreover, we show that differential expression of a considerable fraction of mRNAs and miRNAs is directly attributable to copy number changes of their respective genes. Pathway enrichment analysis on copy number affected mRNAs identified focal adhesion, mTOR signaling, and TGF-beta signaling as altered pathways in HPV-induced carcinogenesis. The biological significance of these findings is supported by functional evidence for PITX2, a key regulator of TGF-beta signaling, and novel miRNA-mRNA interactions. Anchorage-independent cell growth is a hallmark of transformation in vitro [14,15]. While loss of appropriate cell-cell and cell-matrix interactions leads to aberrant integrin signaling and eventually detachment-induced cell death (anoikis) in healthy epithelial cells, cancerous epithelial cells overcome anoikis by epithelial-to-mesenchymal transition (EMT), activation of survival and proliferation pathways, or temporary dormancy [25]. Strikingly, the focal adhesion, mTOR signaling, and TGF-beta signaling pathways identified in present study are implicated in anoikis resistance and induction of EMT [18,26].

TGFB1 has been reported to exert a tumor inhibiting function in early stages of cervical carcinogenesis, while it plays an oncogenic role at a later stage by promotion of EMT and metastasis, induction of angiogenesis, and escape from immune surveillance [19]. In line with this, expression of TGFB1 was found to be downregulated in cervical precancerous lesions compared to normal epithelium although it is increased in cervical cancers [27,28]. This TGF-beta paradox is not unique to cervical carcinogenesis but has been recognized in many other cancers as well [29]. An analysis of 178 cervical carcinomas by The Cancer Genome Atlas (TCGA) network found recurrent deletions of TGF-beta receptor 2 (TGFBR2, 3p24.1) and SMAD4 (18q21.2) in 36% and 28% of analyzed samples, respectively [30]. In accordance, downregulation of both TGFBR2 and SMAD4 was related to copy number loss in our cell line data (Table S1). Moreover, the TCGA network identified common somatic mutations in the TGF-beta pathway for *TGFBR2*, *SMAD4*, *CREBBP*, and *EP300* [30]. These mutations are of an inactivating nature and are believed to deactivate growth-suppressive and pro-apoptotic functions of TGF-beta, leading to a more tumorigenic phenotype.

In a previous study, we reported that the presence of TGFB1 reduced the growth rates of mortal and early immortal FK16A and FK18B cells, but not of late immortal FK16A and FK18B cells, in a SMAD2/SMAD4-independent fashion [31]. Similarly, Creek and colleagues showed that HPV16-transformed cells become insensitive to TGFB1 with increasing passaging and demonstrated that TGFB1 promotes EMT and cell migration [32,33,34]. A recent integrative screen comparing transcriptome and proteome data of HPV16 E6E7 expressing keratinocytes to non-HPV expressing keratinocytes confirmed that TGFB1 plays a regulatory role in HPV-induced carcinogenesis [35]. Here, our extensive longitudinal pathway analysis identified PITX2 (pituitary homeobox 2) as key regulator of TGF-beta signaling during cervical carcinogenesis. We show that elevated PITX2 expression induces apoptosis and cell cycle arrest, indicating that downregulation of PITX2 is important for the survival of HPV-transformed keratinocytes. In line with our results, PITX2 has been shown to cause cell cycle arrest through accumulation of TP53 and CDKN1A in HeLa cells [20,21]. This is achieved through binding of the HPV E6 protein by PITX2 and the subsequent inhibition of TP53 degradation [20]. Moreover, PITX2 has been described as transcriptional activator of CDKN1A in other tissues such as the dental epithelium and neural stem cells [36,37]. Besides an effect on cell growth, others have observed decreased migration upon PITX2 overexpression [20]. Wei and Adelstein showed that PITX2 activates the Rho GTPases RAC1 and RHOA, causing changes to the actin-myosin cytoskeleton thereby reducing cell adhesion and increasing cell motility [20]. Interestingly, our pathway reconstruction also identified RHOA as PITX2 target, as well as ROCK2 and THBS1, two other genes known to be involved in cell adhesion and motility. Future studies on the involvement of PITX2 in growth inhibition, cell cycle, migration, and invasion during HPV-induced transformation are therefore warranted.

The tumor suppressive role of PITX2 in cervical cancer development is further supported by our previously published whole methylome data showing that the *PITX2* promoter is highly methylated in high passages of our four cell lines but not in primary donor keratinocytes [16]. *PITX2* hypermethylation has been described as a promising prognostic biomarker in cervical cancer as well as breast cancer, head and neck squamous cell carcinoma, and prostate cancer [38,39,40,41].

Besides studying the effect of copy number aberrations on gene expression, the tigaR framework also proved suitable for the identification of temporal miRNA-mRNA interactions [17]. Opposed to BRWD3 and PLXNB2 being downregulated upon overexpression of their validated miRNA interaction partner, FOS expression increased upon miR-221-3p overexpression. This observation is potentially caused by the high ectopic levels of miR-221-3p in our experiments, which may result in an indirect effect of miR-221-3p on FOS expression via other miR-221-3p targets. Under physiological conditions, we do not observe this indirect effect as illustrated by the opposing expression directions of FOS (upregulated) and miR-221-3p (downregulated) over time in our cell line model as well as during progression in cervical tissue specimens. In our cell line model, all investigated target mRNAs were upregulated with increasing passages, suggesting oncogenic roles for these genes. While little is known about the functional involvement of BRWD3 and PECR in human cancers, an enrichment of FOS as part of a heterodimer with JUN has previously been linked to anchorage-independent cell growth in our HPV-transformed cell lines as well as HeLa [42,43]. Moreover, PLXNB2 has been described to act as oncogene in ovarian cancer cells, where a knock-down of PLXNB2 led to decreased cell viability and invasion [44]. We demonstrate that miR-30a-3p and miR-138-5p reduce anchorage-independent cell growth of late passage FK18B cells, indicating their functional role in anchorage independence. Additional studies investigating the exact functional roles of the here identified miRNA-mRNA pairs in cervical carcinogenesis might provide valuable therapeutic targets in the future.

## 4. Materials and Methods

### 4.1. Cell Lines and Clinical Specimens

Establishment and culture of the HPV16- (FK16A and FK16B) and HPV18- (FK18A and FK18B) immortalized keratinocyte cell lines has been described previously [7]. Renal epithelium cell line HEK293 was authenticated by STR testing using the Powerplex16 System (Promega, Leiden, The Netherlands) and cultured as described previously [45]. From all four HPV-immortalized keratinocyte cell lines eight passages (Table 1), including both anchorage-dependent (no shading) and anchorage-independent (grey shading) cells, were selected.

### 4.2. RNA and DNA Isolation

Total RNA was isolated using TRIzol Reagent according to the manufacturer’s instructions (Thermo Fisher Scientific, Bleiswijk, The Netherlands). RNA integrity was determined by gel electrophoresis. Total DNA was isolated by standard proteinase K digestion followed by phenol-chloroform purification [46].

### 4.3. Microarrays for DNA, mRNA, and miRNA Profiling

#### 4.3.1. CGH Arrays

To determine genome-wide chromosomal profiles, DNA was hybridized onto comparative genomic hybridization (CGH) microarrays (SurePrint G3 human CGH microarray 4x180K; Agilent Technologies, Santa Clara, CA, USA) according to the manufacturer’s instructions. CGH microarray data are available from the NCBI Gene Expression Omnibus (GEO; http://www.ncbi.nlm.nih.gov/geo/) through series accession number GSE138724.

#### 4.3.2. mRNA Arrays

Global mRNA expression profiles were generated using whole human genome oligo microarrays (G4112F, mRNA 4 × 44K; Agilent Technologies) following the manufacturer’s instructions. High-resolution mRNA expression data are available from GEO through series accession number GSE138079.

#### 4.3.3. miRNA Arrays

Global miRNA expression profiles were determined using human miRNA microarrays (Sureprint G3 human v16 miRNA 8 × 60K; Agilent Technologies) according to the manufacturer’s instructions. These arrays contain in situ synthesized 60-mer oligonucleotides representing 1205 human miRNAs based on the Sanger miRBase release 16. Microarrays for two passages of FK18A (passage 60 and 92, i.e., T4 and T5) failed subsequent quality control and were excluded from analysis. Microarray data have previously been published and are available from GEO through series accession number GSE78279 [16].

#### 4.3.4. Expression Profiling on Tissue Specimens

To confirm in vivo relevance of our cell line data, miRNA, and mRNA expression was analyzed in microarray data obtained from cervical tissue specimens. Global miRNA profiles were obtained from normal HPV-positive cervical epithelium (n = 10), high-grade precancerous lesions (CIN2/3, n = 18), and squamous cell carcinomas (SCC, n = 10). Data is available from GEO through series accession number GSE30656 [47]. The same tissue specimens were used to determine global mRNA expression profiles using whole human genome oligo microarrays (G4112F, mRNA 4 × 44K; Agilent Technologies). Three CIN2/3 did not pass quality control and were excluded from analysis. High-resolution mRNA expression data obtained from cervical tissue samples are available from GEO through series accession number GSE138080. Tissue samples were used in an anonymous fashion in accordance with the Code for Proper Secondary Use of Human Tissues in the Netherlands as formulated by the Dutch Federation of Medical Scientific Organizations (www.federa.org).

### 4.4. Data Pre-Processing and Analysis

#### 4.4.1. Pre-Processing and Matching

DNA copy number data were pre-processed employing median normalization and segmented using the circular binary segmentation method [48]. mRNA gene expression data were background corrected and between array normalization was performed using the robust quantile method [49,50]. Resulting gene expression intensities were transformed using variance stabilizing transformation [51]. MiRNA gene expression data were processed similarly but without background correction.

Prior to integrative downstream analysis, mRNA and miRNA gene transcripts were matched to DNA copy number data based on the genes’ respective chromosomal locations (genomic build GRCh37/hg19) using the overlapPlus matching procedure described in Van Wieringen et al. [52].

#### 4.4.2. Cluster Analysis

Unsupervised hierarchical clustering was performed per cell line on complete mRNA/miRNA expression profiles to explore the overall similarities and differences in expression patterns using maximum as distance measure. The R-package WECCA was used for unsupervised hierarchical clustering of samples based on their DNA copy number profiles per cell line [53]. All dendrograms were constructed using Ward’s linkage as it yields compact and well-separated clusters.

#### 4.4.3. Differential Expression Analysis

To identify m(i)RNA genes with temporal differential expression and to study the association between gene expression and underlying copy number changes, the methodology presented in Miok et al. was employed [17]. Temporal gene expression analysis was performed with a common spline for the cell lines, to identify consistently altered genes over time at a 5% false-discovery rate (FDR). The same methodology was used to investigate associations between mRNA and miRNA gene expression data over time [17]. From this analysis, significantly associated miRNA-mRNA gene pairs with a negative regression parameter were selected. Identified miRNA-mRNA interactions were verified by three independent databases: RNA22 v2.0, miRDB v5, and TargetScan v7 [22,23,24]. RNA22 v2.0 uses conserved sequence features among miRNAs, so-called patterns, to identify binding site hotspots in the 3’ untranslated region (3’UTR) of mRNAs [22]. The identified putative binding sites are then associated to individual miRNAs independent of their seed sequences. miRDB and TargetScan are both based on target prediction features such as miRNA seed conservation or the mRNA sequence flanking the binding site [23,24]. While the MirTarget2 algorithm employed by miRDB uses a support vector machine framework, TargetScan employs a linear regression model. In both cases, prediction features were selected and weighted based on large experimental datasets.

#### 4.4.4. Network Modeling

For network enrichment analysis, 118 potentially relevant pathways stored in the KEGG repository were considered (Table S1) [54]. Enrichment analysis showed significant overrepresentation of 21 pathways within the copy number related differentially expressed mRNAs (chi-square test with FDR correction). From these 21 pathways, focal adhesion (KEGG hsa04510), TGF-beta signaling (KEGG hsa04350), and mTOR signaling (KEGG hsa04150) were selected for subsequent pathway reconstruction analysis based on the following criteria: (1) total number of genes in pathway is between 50 and 200 (requirement for downstream pathway reconstruction), (2) more than 5% of genes within the pathway showed copy number induced differential expression. Although the B-cell receptor signaling pathway (hsa04662) and cell cycle (hsa04110) also met these criteria, they were not investigated further. This analysis aimed to identify hub genes, which represent possible key regulators of the pathway in the given context. For this purpose, mRNA gene expression data from the experiment was mapped to the pathways as defined by the KEGG repository and the vector autoregressive model with time-varying DNA copy number was employed [54,55]. This model allows to identify both temporal (signals propagated over time) and contemporaneous (signal propagated among the genes) interactions among the genes. This integrative methodology allows identification of the ‘within-level’ relationship in the mRNA gene levels as well as relations between different molecular levels [55,56].

### 4.5. Quantitative Reverse Transcription-PCR (qRT-PCR)

#### 4.5.1. mRNA qRT-PCR

To determine expression levels of BRWD3, DEK, DKK3, FOS, PECR, PLXNB2, SNRPA, and SLC25A36, cDNA was synthesized from 200 ng total RNA template using specific reverse primers (Table S6a). All primer pairs were intron-flanking with an exception for mono-exonic FOS. For analysis of FOS expression, RNA samples were therefore treated with RQ1 DNase (Promega) prior to reverse transcription. The resulting cDNA was used for SYBR Green qPCR on the ABI7500 Fast Real-Time PCR System (Thermo Fisher Scientific). Specificity of the PCR reaction was determined generating melting curves for each reaction. Each 25 µL PCR reaction contained 12.5 µL 2× SYBR Green master mix (Thermo Fisher Scientific), 0.5 µM forward and reverse primers, and 2.5 µL cDNA. Cycle conditions used were according to the manufacturer’s instructions. Data were normalized so SNRPA using the 2^−Δ*C*t^ method [57].

#### 4.5.2. MiRNA qRT-PCR

Expression of miR-100-5p, miR-103a-3p, miR-125b-5p, miR-138-5p, miR-15b-5p, miR-21-3p, miR-221-3p, miR-221-5p, and U75 were measured using TaqMan microRNA assays (000437, 000439, 000449, 002284, 000390, 002438, 000524, 002096, 001219; Thermo Fisher Scientific). The TaqMan microRNA Reverse Transcription kit (Thermo Fisher Scientific) was used for cDNA synthesis from 10 ng total RNA template according to the manufacturer’s instructions. Quantitative PCR reactions were performed on the ABI7500 Fast Real-Time PCR System (Thermo Fisher Scientific). Each 10 µL qPCR reaction contained 5 µL TaqMan^®^ Universal Master Mix II (Thermo Fisher Scientific), 0.5 µL miRNA specific TaqMan assays, 3.5 µL H2O, and 1 µL cDNA. Cycle conditions for cDNA synthesis and PCR were used according to the manufacturer’s protocols. miRNA expression data were normalized to U75 using the 2^−Δ*C*t^ method [57].

### 4.6. Viral Transduction and Flow Cytometry

The coding sequence of PITX2 transcript variant 2, amplified by Phusion High-Fidelity PCR (New England Biolabs (NEB), Ipswich, MA, USA), was cloned into the lentiviral vector LeGO-iG2 [58] (Addgene, Teddington, UK) via the pcDNA3.1(+) Mammalian Expression Vector (Thermo Fisher Scientific) using HindIII and NotI restriction sites. For primer sequences please refer to Table S6b. Restriction enzymes were purchased from NEB. For virus production, HEK293 cells were transfected with lentiviral packaging plasmids pRSV-rev, pCMV-VSV-G, and pMDLg/RRE together with LeGO-iG2 empty vector or LeGO-iG2-PITX2 using polyethylenimine (PEI; Sigma-Aldrich, St. Louis, MO, USA). Late passage FK18B cells (ca. passage 190) were transduced with lentivirus using polybrene (Thermo Fisher Scientific) for 24 h. Cells were harvested for RNA 72 h after transduction. Analysis of GFP expression was performed on a FACS-Calibur flow cytometer (BD Biosciences, Breda, The Netherlands) equipped with the CellQuest Pro data acquisition and analysis software. Two independent experiments were performed.

### 4.7. Protein Lysis and Western Blot Analysis

Whole cell lysates were prepared using the RIPA Lysis Buffer System (Santa Cruz Biotechnology, Dallas, TX, USA) with benzonase (Sigma-Aldrich) 72 h after transduction. Twenty-five micrograms of protein was fractionated on 8–16% precast polyacrylamide gels (Bio-Rad, Hercules, CA, USA) and transferred to a nitrocellulose membrane. Western blots were incubated with Anti-ACTB (1:1000, rabbit, Cell Signaling Technologies, Danvers, MA, USA), Anti-Cleaved CASP3 (1:1000, rabbit, BD Biosciences), Anti-p21 (CDKN1A; 1:1000, mouse, Sigma-Aldrich), Anti-p27 (CDKN1B; 1:1000, mouse, BD Biosciences), Anti-PCNA (1:1000, mouse, Dako), Anti-PITX2 (1:1000, rabbit, Abcam, Cambridge, UK), or Anti-TP53 (1:1000, mouse, Dako, Glostrup, Denmark). Antibody binding was visualized using goat-α-rabbit IRDye 680RD and goat-α-mouse IRDye 800CW (both LI-COR Biosciences, Lincoln, NE, USA). As positive control for TP53/CDKN1A signaling, FK18B cells treated with 50 µM etoposide (Sigma-Aldrich) for three hours were included in the Western blot.

### 4.8. MiRNA Transfection and Luciferase Dual-Reporter Assays

Late passage FK18B cells (ca. passage 190) were transiently transfected with 30nM miRIDIAN microRNA mimics for miR-138-5p, miR-21-3p, miR-221-3p, and negative control #2 (C-300605-05, C-301023-01, C-300578-05, CN-002000-01; Horizon Discovery, Cambridge, UK) using Dharmafect 1 (Horizon Discovery) for 22 h according to the manufacturer’s instructions. Cells were harvested for RNA 48 h after transfection. Each experiment was carried out three times.

The predicted 3’UTR binding sites of BRWD3, FOS, PECR, and PLXNB2 amplified by Phusion High-Fidelity PCR (NEB) were cloned into the pmirGLO Dual-Luciferase miRNA Target Expression Vector (Promega) using the SacI (NEB) and XhoI (NEB) restriction sites. Predicted miRNA binding sites were mutated using the Q5 Site-Directed Mutagenesis Kit (NEB) according to the manufacturer’s instructions (Figure 4a–d). Primer sequences are listed in Table S6c–d. HEK293 were seeded in triplicate in 96-well plates (7500 cells/well) and co-transfected with 6.67 nM mimics and 20 ng pmiRGLO vector the following day. Firefly luciferase activity was measured using the Dual-Glo Luciferase Assay (Promega) according to the manufacturer’s instructions 48 h after transfection. *Renilla* luciferase activity was determined as internal control and used for normalization. Each experiment was carried out three times.

### 4.9. MiRNA Transfection and Cell Viability Assays

Late passage FK18B cells (passage 220) were transfected with miRNA mimics using a reverse transfection method. Briefly, the miRNA mimic/transfection reagent complexes were made by adding miRNA mimics for miR-138-5p, miR-21-3p, miR-221-3p, negative control #1, and UBB siRNA (positive control; C-300605-05, C-301023-01, C-300578-05, CN-001000-01, L-003290-00; Horizon Discovery) and Dharmafect 4 transfection reagent (Horizon Discovery) to tissue culture coated plates and ultra-low attachment plates (Corning, New York, NY, USA). Subsequently, cells were added and cell viability was measured after 72 h of incubation using the luminescent CellTiter-GLO 3D assay (Promega) according to the manufacturer’s protocol. Each experiment was carried out three times.

## 5. Conclusions

In conclusion, we present a unique longitudinal multi-level dataset on HPV-induced transformation. Using state-of-the-art analysis tools, we identified key pathway regulators and novel miRNA-mRNA interactions relevant to the carcinogenic process. Our results highlight the importance of chromosomal alterations in the acquisition of anchorage independence during carcinogenesis. Additional functional studies on the identified key pathway regulators and differentially expressed m(i)RNAs might yield potential therapeutic targets and disease markers in the future.

## Figures and Tables

**Table 1:** Passage numbers included for all four HPV-transformed cell lines [16].

Time Point	FK16A	FK16B	FK18A	FK18B
T1	p18	p21	p19	p17
T2	p22	p22	p21	p18
T3	p39	p45	p47	p40
T4	p52	p51	p60	p52
T5	p109	p89	p92	p90
T6	p115	p102	p99	p98
T7	p206	p140	p148	p146
T8	p222	p169	p160	p164

FK16A and FK16B contain HPV16, whereas FK18A and FK18B contain HPV18. No shading: anchorage-dependent cells; grey shading: anchorage-independent cells.

**Table 2:** Key regulators of selected pathways.

(a) Focal Adhesion			(b) mTOR Signaling		
Regulator	Number of Regulated Genes	Regulated Genes in Pathway (%)	Regulator	Number of Regulated Genes	Regulated Genes in Pathway (%)
TNN	72	36.0	RPS6KA2	18	34.6
LAMA4	40	20.0	IGF1	16	30.8
COL5A3	32	16.0	HIF1A	12	23.1
MYLPF	30	15.0	PRKAA2	4	7.7
MYL10	26	13.0	DDIT4	3	5.8
FYN	24	12.0	STK11	3	5.8
PDGFD	22	11.0	EIF4B	3	5.8
ITGA4	21	10.5	PIK3CD	2	3.8
COMP	17	8.5	PIK3R3	2	3.8
LAMA3	16	8.0	VEGFC	2	3.8
**(c) TGF-Beta Signaling**					
**Regulator**	**Number of Regulated Genes**	**Regulated Genes in Pathway (%)**			
PITX2	45	52.9			
ID4	34	40.0			
LEFTY2	24	28.2			
INHBA	22	25.9			
BMP4	19	22.4			
ID1	18	21.2			
FST	15	17.6			
AMHR2	10	11.8			
COMP	10	11.8			
TGFB2	7	8.2			

## Supplementary Materials

The following are available online at https://www.mdpi.com/2072-6694/12/3/700/s1, Figure S1: Technical validation of microarray results by qRT-PCR, Table S1: mRNAs that consistently change over time in at least three out of four cell lines, Table S2: miRNAs that consistently change over time in at least three out of four cell lines, Table S3: Network enrichment analysis of copy number related genes in 118 KEGG pathways, Table S4: Key regulators of selected pathways and their interactions, Table S5: Predicted mRNA-miRNA interactions, Table S6: Primer sequences.
