# Supplementary material for: Identification of Deregulated Pathways, Key Regulators, and Novel miRNA-mRNA Interactions in HPV-Mediated Transformation
Source: Cancers (Basel). 2020 Mar 16;12(3):700. doi: 10.3390/cancers12030700 (PMC7140059; doi:10.3390/cancers12030700)
Supplement: Supplementary file 1 [file cancers-12-00700-s001.zip › cancers-735842-suppl-proofreading/cancers-735842- Figure S1.pdf]

Supplementary Materials: Identification of Deregulated Pathways, Key Regulators, and Novel miRNA-mRNA Interactions in HPV-Mediated Transformation

Iris Babion, Viktorian Miok , Annelieke Jaspers, Angelina Huseinovic, Renske D.M. Steenbergen, Wessel N. van Wieringen and Saskia M. Wilting

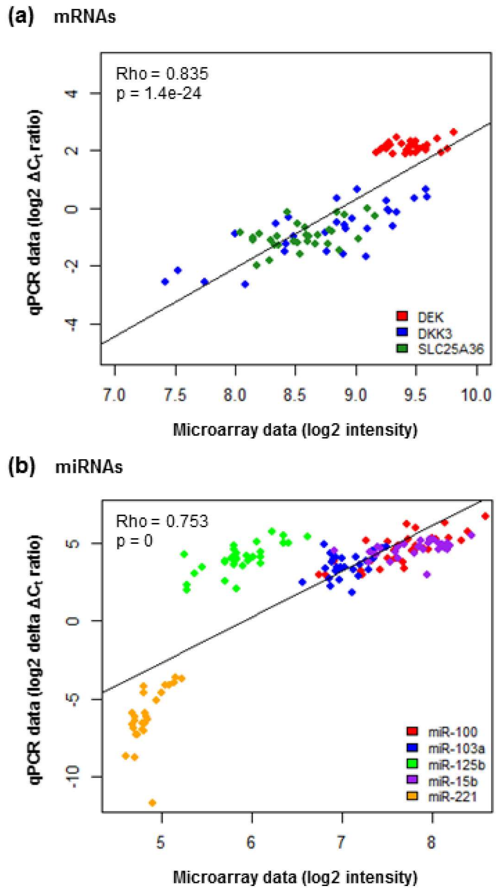

Figure S1. Technical validation of microarray results by qRT-PCR. (**a**) mRNAs DEK, DKK3, SLC25A36, and (**b**) miRNAs miR-100-5p, miR-103a-3p, miR-125b-5p, miR-15b-5p, and miR-221-5p. The Spearman correlation coefficient (Rho) and associated p-value (p) are indicated.

© 2020 by the authors. Licensee MDPI, Basel, Switzerland. This article is an open access article distributed under the terms and conditions of the Creative Commons Attribution (CC BY) license (http://creativecommons.org/licenses/by/4.0/).
